# Ensemble perception during multiple-object tracking

**DOI:** 10.3758/s13414-020-02219-4

**Published:** 2021-01-06

**Authors:** Reem Alzahabi, Matthew S. Cain

**Affiliations:** 1 Center for Applied Brain and Cognitive Sciences, Tufts University, Medford, MA 02155 USA; 2 US Army Combat Capabilities Development Command Soldier Center, Natick, MA USA

**Keywords:** Grouping and segmentation, Visual perception, Attention

## Abstract

Multiple-object tracking studies consistently reveal attentive tracking limits of approximately three to five items. How do factors such as visual grouping and ensemble perception impact these capacity limits? Which heuristics lead to the perception of multiple objects as a group? This work investigates the role of grouping on multiple-object tracking ability, and more specifically, in identifying the heuristics that lead to the formation and perception of ensembles within dynamic contexts. First, we show that group tracking limits are approximately four groups of objects and are independent of the number of items that compose the groups. Further, we show that group tracking performance declines as inter-object spacing increases. We also demonstrate the role of group rigidity in tracking performance in that disruptions to common fate negatively impact ensemble tracking ability. The findings from this work contribute to our overall understanding of the perception of dynamic groups of objects. They characterize the properties that determine the formation and perception of dynamic object ensembles. In addition, they inform development and design decisions considering cognitive limitations involving tracking groups of objects.

## Introduction

Attention allows for humans to make sense of a complex environment by selecting a subset of information for further processing (Allport, 1989). Attending to objects in the environment becomes more difficult when the objects are in motion. Even so, in a dynamic visual world, humans have the ability to simultaneously track multiple independent moving objects. For instance, while viewing a sports game, it is entirely possible for a viewer to keep track of multiple players as they make their way to different positions across the field. Research on multiple-object tracking provides evidence that attention can be simultaneously deployed to multiple distinct object locations and this operation can be carried out in parallel across several independent locations in the visual field (Cavanagh & Alvarez, 2005).

Since the emergence of multiple-object tracking (MOT) as an active area of research, we have gained an understanding of the mechanisms that support the tracking of multiple objects and the limitations of this ability. The typical paradigm used to examine tracking ability involves asking participants to track a number of targets as they move randomly amongst distractor items. The display usually consists of identical items (distractors and targets) that move within a defined area while bouncing off one another and the display border. After a tracking period (approximately 10 s), participants are asked to indicate the target items either through a probe method (target yes/no), or by selecting as many targets as they are able to.

Generally, an attentive tracking limit of approximately three to five items emerges (Pylyshyn & Storm,^1988^). However, this tracking limit is not entirely fixed, but, rather, is subject to several factors. For instance, speed impacts one’s ability to track moving objects such that one can track up to eight objects at slow speeds, but only one object at fast speeds (Alvarez & Franconeri, 2007). Other variables such as stimulus complexity (Horowitz et al., 2007), depth plane in which stimuli are viewed (Viswanathan & Mingolla, 2002), individual differences (e.g., visual short-term memory; Oksama & Hyona, 2004), and self-motion (Thomas & Seiffert, 2010) also play a role in one’s ability to track multiple moving objects. Research on the mechanisms of multiple-object tracking suggests a parallel account of attentional deployment, such that multiple objects are simultaneously, rather than serially, attended to during tracking (Howe et al., 2010). Furthermore, similar to other cognitive abilities that are amenable to change, expertise impacts tracking capacity. One study reported that air traffic controllers exhibit exceptional tracking skills (Allen et al., 2004). Other domains that afford extensive practice, such as team sports, video game play, and military activities are also thought to play a role in improving tracking ability (Cavanagh & Alvarez, 2005; Green & Bavelier, 2006).

While research on classic multiple-object tracking has become well developed, there remains a gap in our understanding of how grouping plays a role in multiple-object tracking. In reality, our visual experiences extend beyond perceiving discrete objects. Take for example a flock of birds overhead. Or, for instance, a group of autonomous robots. In both of these examples, qualities such as common fate give rise to objects being perceived as a group. Although the objects are not rigidly connected, they may or may not be perceived as a group by the visual system, depending on the grouping features present. More generally, the visual world consists of scenes that are organized into perceptual groups defined by the traditional Gestalt principles of similarity, proximity, common fate, etc. (Palmer, 1999; Wagemans et al., 2012).

Perceptual grouping is a process in which image elements are aggregated into larger collections (Feldman, 1999) and is thought to be automatic and preattentive (Kahneman & Henik, 1981; Prinzmetal & Banks, 1977; Treisman, 1982). While human perceivers easily and rapidly group and perceptually organize based on a scene’s “most reasonable” interpretation, grouping has long been a computationally difficult problem in vision science. Formalizing the parameters that make a “good” group is difficult without an objective physical definition, unlike other perceptual variables such as depth, color, or motion (Feldman, 1999). Work on ensemble perception suggests that the human visual system can quickly and easily compute summary statistics of a group of objects, such as speed, orientation, or brightness (Whitney & Yamanashi Leib, 2018). Further, other studies have shown that observers can successfully extract information on multiple ensembles, although undivided attention to an ensemble results in more enhanced and in-depth processing (Attarha & Moore, 2015).

To date, the literatures on multiple-object tracking and grouping have progressed along independent trajectories and there remains a lack of research on the tracking of multiple ensembles. In the context of multiple-object tracking, do the same constraints that apply to tracking individualized objects also apply to tracking groups of objects? It has been suggested that “object-based” attention and “group-based” attention may reflect the operation of the same underlying attentional circuits (Scholl, 2001). Attention spreads across an entire group in which it falls (Driver & Baylis, 1998). Also, attention is more easily moved within a perceptual group, compared to movement between groups, suggesting groups share object-like qualities (Feldman, 1999). Thus, the primary goal of this investigation was to characterize how MOT applies to groups of objects as well as characterize the qualities that give rise to the perception of multiple objects as a group in a dynamic context.

While research on grouping within the context of multiple-object tracking is lacking, a few studies report findings related to objecthood and grouping. One study reports benefits of perceptual grouping on visual working memory (VWM) such that VWM, which is limited in capacity, can be facilitated by Gestalt principles of grouping (Li, Qian, & Liang, 2018). Specifically, change detection accuracy was found to be better for grouped items compared to ungrouped items. In addition, memory performance improved for grouped items and this improvement was not significantly different from the effect of grouping by physical connectedness. Early work by Yantis (1992) investigated the tracking of objects that fell within a group configuration, which he termed “multielement visual tracking.” Across seven experiments, manipulating the extent to which perceptual grouping was possible impacted tracking performance. Grouping targets into a virtual polygon improved tracking accuracy and common fate factors (both rigid and non-rigid constraints) impacted how successfully a perceptual group could be maintained during tracking. Merkel, Hopf, and Shoenfeld (2017) provide evidence that tracked items can be grouped together by an illusory contour and object-based attention is deployed throughout the tracking process. Furthermore, work by Suganuma and Yokosawa (2006) investigated characteristics of motion within the context of MOT and found that there are impairments in tracking ability when motion of target and distractor items share properties. These studies suggest that there are grouping qualities that impact the perception of multiple objects during memory and tracking.

Another line of work considering objecthood within the context of MOT investigates the specific units that are tracked during MOT (Scholl, Pylyshyn, & Feldman, 2001). Research suggests that the unit for tracking is an “object,” but what exactly is an object in MOT? In this work, the general finding was consistent with other studies in that people could successfully track approximately four objects. However, people were unable to track these same four targets – with the same target locations and target/distractor trajectories – when they were parts of larger bar-shaped objects. The authors argue for the case of object merging, in which whole-object tracking is obligatory, and parts of objects cannot be tracked. This work provides evidence for object-based tracking in that attention must be allocated to objects, rather than an arbitrary collection of features. In regards to the current investigation, the question remains as to whether groups of objects are simply multi-element objects, or if groups of objects have qualities that exclude them from being perceived as “objects.”

The second goal of the current investigation was to understand the limitations of humans’ ability to track multiple groups of objects. In addition, we sought to characterize the configural properties of a scene that allow one to track multiple groups of objects. More specifically, how does grouping impact multiple-object tracking capacity? What are the constraints to the perception of ensembles within dynamic contexts? How can we characterize objecthood and group belongingness within the context of MOT? In the sections that follow, we report a series of experiments investigating the heuristics of organization that guide the perception of groups of objects, in addition to the flexibility of the boundaries of these heuristics. In sum, our aim is to characterize the factors that are involved in the formation and perception of moving groups of objects. Three primary questions guide the current study: (1) Is tracking capacity impacted by the number of items the group is composed of? (2) To what extent do inter-object spacing and common fate contribute to the perception of moving groups of objects? (3) Does the perception of common fate rely on the rigidity of the group? That is, to what extent can the individuated objects within a group deviate from the group’s overall motion and the group still be perceived as a group?

## General methodology

**Fig. 1:**
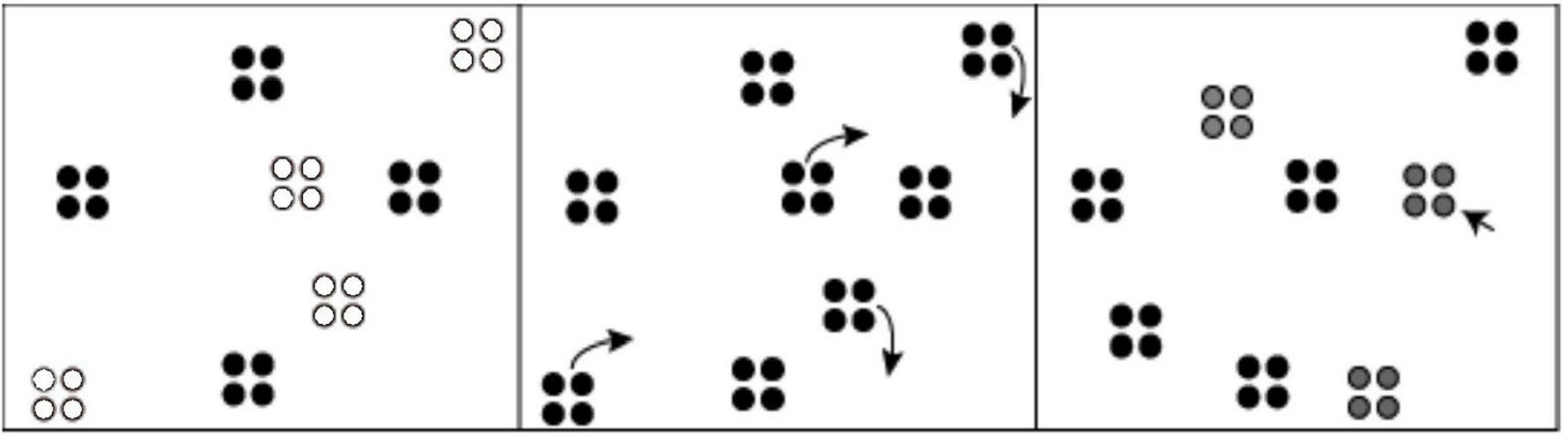
Depiction of the general multiple-object tracking experimental design. Target groups were highlighted for 2 s (here, the highlight is indicated by white-colored dots, in the actual experiment, the dots flashed), followed by a 7-s tracking phase. Participants then selected the target groups (Experiment 1)/ dots (Experiments 2 and 3) using the mouse cursor. Note variations in the group configurations for Experiments 2 and 3 as described in the text

Experiments consisted of a series of multiple-object tracking tasks. Participants tracked groups of objects as they moved among groups of distractors (see Fig. 1). Stimuli consisted of black dots presented on a white screen. Each group of dots moved in synchronous motion following a random independent trajectory and at a speed of 3 ^o^/s. Target groups, indicated with a flash at the start of each trial, moved among distractor groups. After the tracking period, participants were probed for the identity of the target groups. In each experiment, participants were provided with feedback on their performance in the practice trials, such that incorrectly selected groups appeared in red and correctly selected groups appeared in green. This feedback was absent in the experimental trials. Block order for each experiment was randomized across participants. Details of each block of trials are explained in the detailed methodology below. Tracking displays were programmed in Unity and presented on a 1,920 x 1,080 pixel, 60-Hz monitor controlled by a Dell Optiplex 9020 computer. Viewing distance was approximately 57 cm.

After completing the multiple-object tracking task, participants completed surveys including a demographics questionnaire, Media Use Questionnaire (Baumgartner et al., 2017), Video Game Experience Questionnaire (Donohue et al., 2010), Brief Sensation Seeking Scale (Stephenson, et al., 2003), Barratt Impulsiveness Scale (Patton, Stanford, & Barratt, 1995), and the State/Trait Anxiety Inventory (Spielberger, 1989). These surveys were completed as part of lab baseline data collection and are not analyzed in the current study. Participants participated for monetary compensation and provided informed consent in accordance with the policies of the Tufts University Social, Behavioral, and Educational Research Institutional Review Board. They were offered a debriefing form upon completion of the experiment. All experiments were conducted using the same equipment and procedural setup, with variations, as noted in each experiment.

## Experiment 1

The goal of the first experiment was to understand the capacity limitations applicable to tracking multiple groups of objects. Thus far, our general understanding of tracking objects in our environment has been based on independent, single objects. Specifically, attentive tracking is limited to approximately three to five items (Pylyshyn & Storm, 1988), similar to the number of items one can hold in working memory – about four objects (Cowan, 2001). Here, we are interested in understanding how grouping and ensemble perception impact multiple-object tracking capacity. We want to identify if tracking groups of objects falls within the same capacity limitations as independent objects. In addition, we want to identify whether the size of the group (i.e., the number of items the group is composed of) impacts tracking capacity. Do individuals perceive groups of objects holistically? Is a group of two objects perceived in the same way that a group of eight objects is?

If group perception is non-holistic, we would observe that individuals can successfully track, on average, four objects, regardless of their group configuration. Alternatively, if group perception is holistic, we would observe that individuals can successfully track four groups of objects, on average. This capacity may or may not be mediated by group size. It is possible that groups of, for example, two objects or eight objects, are perceived similarly such that the objects in both groups are configured as one group. Alternatively, larger group sizes might be configured into chunks of multiple separate groups. This account would indicate that larger group sizes require more mental capacity for tracking than smaller group sizes. In contrast, it is possible that larger group sizes require less capacity for tracking, in that there are multiple target points within the holistic group to keep track of.

### Participants

Thirty-two participants (25 females; mean age = 22.59 years) participated in Experiment 1.

### Stimuli and procedure

Experiment 1 consisted of a manipulation of the size (number of items) of the target and distractor groups to examine the impact that group size has on ensemble perception and tracking. Participants completed 18 blocks of 16 trials each, yielding 288 total experimental trials. Pilot data indicated low variability in performance across each block of trials, informing our decision to cap blocks at 16 trials each. Eighteen practice trials, representing group configurations from each block, preceded the experimental trials.

**Fig. 2:**
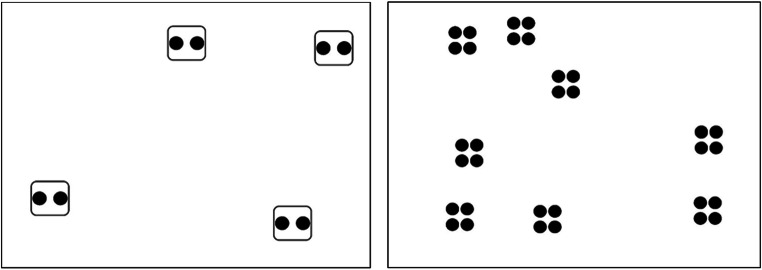
Stimuli for Experiment 1. Left panel presents a trial of group size 2, two groups to track, and enclosed groups. Right panel presents a trial of group size 4, four groups to track, and non-enclosed groups

Three factors varied across blocks: group size (two, four, or eight objects), number of groups to track (two, four, or six), and enclosure status (yes or no) (see Figs. 1 and 2). This resulted in a tracking load that ranged from four objects total (two groups x two objects in each group) to 48 objects total (six groups x eight objects in each group). Physically enclosed groups served as a control condition, allowing comparisons between grouping that is perceptually defined versus physical grouping. In each block, group size, number of groups, and enclosure status was identical for target and distractor groups.

Groups were composed of clusters of dots and the visual angle of each group was held constant (approximately 2^o^ × 2^o^), regardless of group size. As such, dot size varied based on the size of the group (0.35^o^ radius for group size 2, 0.30^o^ radius for group size 4, and 0.2^o^ for group size 8). Enclosures were 2^o^ x 2^o^ squares surrounding the dot cluster.

Participants were asked to perform the tracking task and were informed that the size of the groups would change across blocks. They were specifically told that they “would see groups of dots, and would be asked to track some of the groups,” while “the number of dots in each group would change” across blocks of trials. Initial positions of the groups were randomly chosen, with the constraint that groups did not overlap with one another or with the frame’s border. The boundaries of the frame coincided with the dimensions of the screen used to display the experiment. At the beginning of each trial, a subset of groups was flashed (2 s) in gray, indicating their status as target groups. All groups moved randomly around the display for 7 s, avoiding each other and remaining within the frame’s border. Random motion of each dot group was generated by assigning a random two-dimensional (2D) vector to each dot group, followed by an acceleration phase. If two groups of dots were too close to one another, then a repulsive force changed the collision between the two dot groups. That repulsive force had a direction that was aligned with the centers of the moving dot groups that had neared collision. At the end of each trial, all groups stopped moving and the mouse pointer appeared, allowing participants to select their choice of target groups. Upon selecting one dot, the entire group would appear as selected, which was indicated as a change to a gray color. After selecting a number of groups that was consistent with the number of target groups for that particular trial, the experiment proceeded to the next trial.

### Experiment 1: Results and discussion

**Fig. 3:**
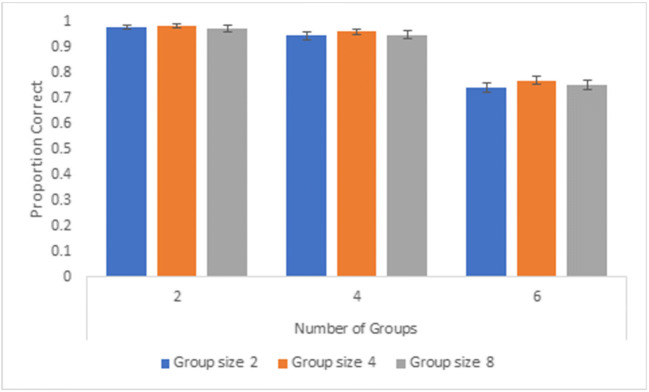
Overall proportion correct across participants for Experiment 1, non-enclosed groups. Error bars denote standard errors of the means

**Fig. 4:**
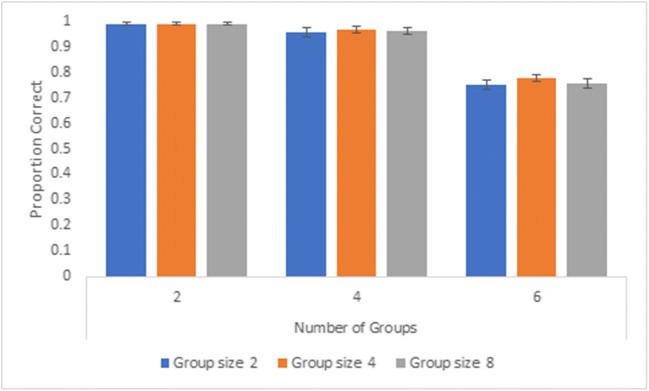
Overall proportion correct across participants for Experiment 1, enclosed groups. Error bars denote standard errors of the means

We calculated the average proportion of correct responses for each group size (two, four, eight) and number of groups (two, four, six). Overall, tracking performance for two groups (mean accuracy = 98.5%, SD = 3.4%) and four groups (mean accuracy = 95.7%, SD = 8.2%) was very high, regardless of the number of items the group is composed of and whether the groups were enclosed or not. Overall accuracy declined to 75.8% (SD = 8.6%) for tracking of six groups. Tracking performance across all numbers of groups and enclosures for group size 2 averaged 89.5% (SD = 6.4%), 90.8% (SD = 4.7%) for group size 4, and 89.7% (SD = 6.4%) for group size 8. A 3 (group size: two, four, eight) x 3 (number of groups: two, four, six) x 2 (enclosure status: yes, no) repeated-measures analysis of variance (ANOVA) revealed significant main effects of enclosure status, *F*(1, 31) = 4.30, *p* = 0.047, partial η^2^ = 0.12, group size, *F*(2,62) = 4.95, *p* = 0.01, partial η^2^ = 0.14, and number of groups, *F*(2, 62) = 176.87, *p* < 0.001, partial η^2^ = 0.85. All interactions were non-significant, ps > 0.16. A repeated-measures ANOVA for non-enclosed groups revealed significant main effects of group size, *F*(2, 62) = 5.08, *p* = 0.01, partial η^2^ = 0.14, and number of groups, *F*(2, 62) = 165.50, *p* < 0.001, partial η^2^ = 0.84 and a non-significant interaction, *F*(4, 124) = 0.67, *p* = 0.61, partial η^2^ = 0.02 (see Fig. 3). For enclosed groups, the effect of number of groups was significant, *F*(2, 62) = 165.81, *p* < 0.001, partial η^2^ = 0.84, while the main effect of group size, *F*(2, 62) = 2.31, *p* = 0.11, partial η^2^ = 0.07, and the interaction, *F*(4, 124) = 1.39, *p* = 0.24, partial η^2^ = 0.04, were not significant. (See Fig. 4). Generally, tracking performance for enclosed groups did not vary as a function of group size but was impacted by the number of groups required to track.

Based on these findings, groups of objects were perceived similarly to individuated objects, indicated by capacity limits for tracking groups that are similar to tracking objects. Further, people treated groups of objects, within the context of MOT, as if they were objects, and the perception of groups of objects that were physically enclosed was not impacted by the size (number of items) of the group. In addition, the enclosure manipulation had a significant effect, demonstrating that physical enclosure is a property that encourages granting objecthood to groups of individuated objects. In the following experiments, we tested other properties’ contribution to group perception. Experiment 2 delves into spacing qualities that lead groups of objects to be perceived as a group. That is, when does the perception of a group of objects begin to break down such that objects are perceived individually? Can we better define what a group is in regards to inter-object spacing?

## Experiment 2

The goal of the second experiment is to understand the extent to which the formation of groups is driven by inter-object spacing. More specifically, what are the limits of inter-object spacing so that objects are perceived as a group? Here, we consider four different extents of object spacing ranging from relatively “close” to “far” (description of the specific spacings described in *Methods* below). Groups were rigidly formed and followed a common fate motion trajectory. The extent of spacing was determined by the common region that the group occupied and the proximity of the individual objects to one another. If group perception is not contingent on inter-object spacing, then we should observe equivalent tracking performance across all extents of spacing given consistent common fate. Alternatively, if group perception is contingent on both common fate and spacing, then we would observe that tracking performance declines as spacing increases.

### Participants

Forty participants (29 females; mean age = 22.29 years) participated in Experiment 2. Three participants were excluded for having accuracy below 50% (mean dot accuracy = 33.3%).

### Stimuli and procedure

Experiment 2 consisted of a manipulation of the spacing between group objects to examine the impact that inter-object spacing has on group perception and tracking. Participants completed four blocks of 16 trials each, yielding 64 total experimental trials. Four practice trials, representing spacing configurations from each block, preceded the experimental trials.

**Fig. 5:**
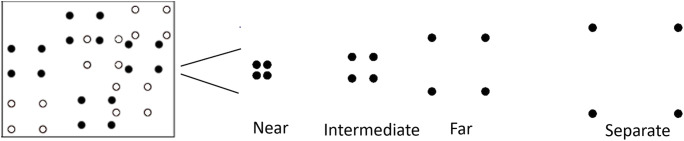
Stimuli for Experiment 2. Schematic representation of the near, intermediate, far, and separate group spacings. White-colored dots did not appear in the actual experiment but are used for representation of groups in the figure

The primary factor that varied across blocks was the spacing between the dots (see Fig. 5). One block consisted of “near” spacing, which reflected inter-object spacing identical to Experiment 1. The other blocks consisted of increasingly spaced dots within each group: “intermediate,” “far,” and “separate” spacings. Dots within each group were confined within an imaginary square with the following edge lengths (in visual angle): Near = 2.01^o^, Intermediate = 3.22^o^, Far = 6.43^o^, Separate = 10.03^o^. Groups had a rigid formation, such that the dots in each group had common fate and moved in uniform motion trajectories. Within each block, inter-object spacing was identical for target and distractor groups. To account for any confounds between intra-group distances and moving distances, we implemented motion constraints for the movement of the groups such that groups could overlap with one another, but individual dots could not. Unlike Experiment 1, the group size and number of target/distractor groups did not vary across blocks, such that all blocks consisted of groups composed of four dots, and participants were always required to track four target groups amongst four distractor groups. Also, no groups were enclosed. Dot size remained constant at 0.30^o^ radius across all blocks.

Participants were asked to perform the tracking task and were explicitly told to track four groups of dots. They were informed that the arrangement of the dots in each group would change across blocks and were asked to select 16 dots at probe (four dots in each group x four groups). Generation of group’s motion was similar to Experiment 1, with the exception that there was no constraint to the groups overlapping with one another. Targets were identified and tracking ensued in a manner identical to Experiment 1. At the end of each trial, all dots stopped moving and the mouse pointer appeared, allowing participants to select their choice of target dots. Participants selected a total of 16 dots, and when selected, the dot would change to a gray color. After selecting 16 dots during probe, the experiment proceeded to the next trial.

### Experiment 2: Results and discussion

**Fig. 6:**
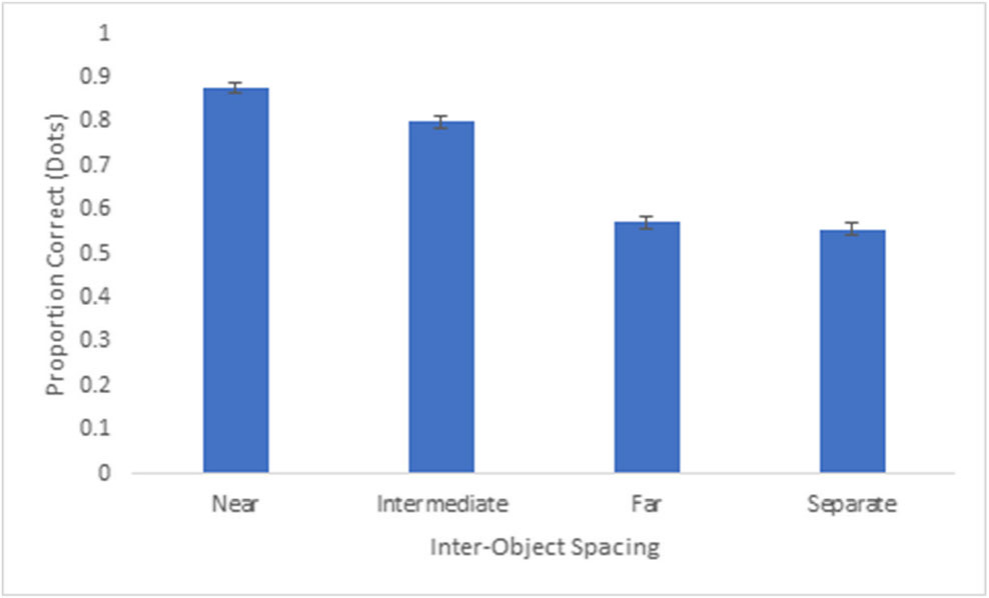
Overall proportion correct (dots selected) across participants for Experiment 2. Error bars denote standard errors of the means

**Fig. 7:**
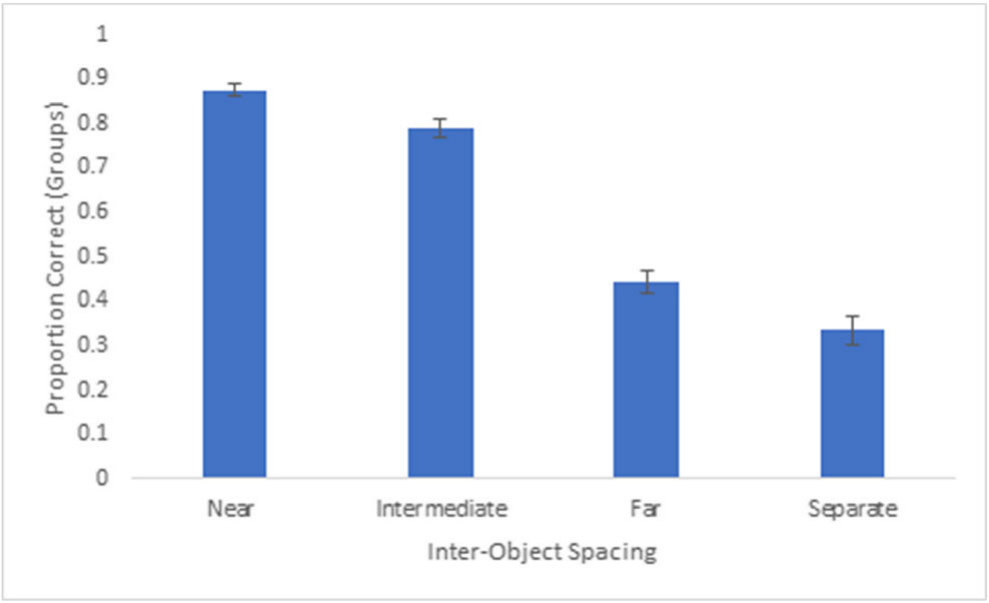
Overall proportion correct groups (all four dots selected) across participants for Experiment 2. Error bars denote standard errors of the means

We calculated the average proportion of correct responses for each extent of inter-object spacing based on both dot and group responses. Dot accuracy was defined as the number of correctly selected dots on each trial, from a total of 16 dots. Group accuracy was defined as the number groups correctly selected in their entirety (i.e., all four dots in a group). An analysis of variance revealed a main effect of inter-object spacing for both dot accuracy, F(3,111) = 292.86, p < 0.001, partial η^2^ = 0.89, and group accuracy, F(3,111) = 231.0, p < 0.001, partial η^2^ = 0.87 (see Figs. 6 and 7). Generally, we observed a gradual decline in tracking performance across increased spacings, and tracking performance was nearly at or below chance for far and separate spacings. Patterns in the dot accuracy and group accuracy data were comparable, and to provide a more parsimonious set of results we focused our follow-up analyses on the group accuracy data. Group accuracy was highest for near spacing (M = 87.5%) and was significantly higher than the intermediate spacing (M = 79.0%), t(37) = 6.70, p < 0.001. Intermediate spacing group accuracy was significantly higher than far group spacing (M = 44.2%), t(37) = 15.86, p < 0.001, which was also significantly higher than separate group spacing (M = 33.4%), t(37) = 4.64, p < 0.001.

**Fig. 8:**
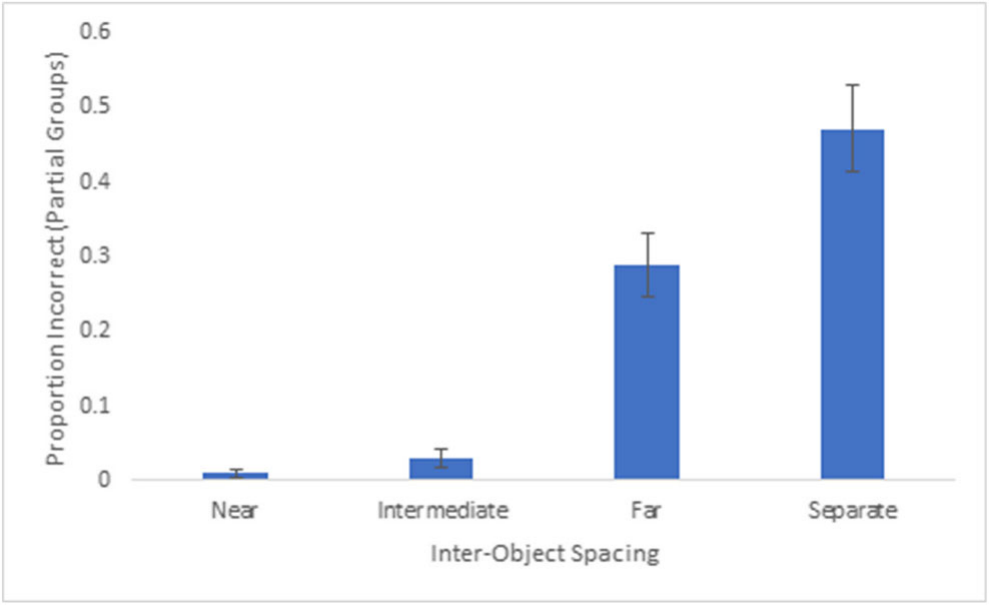
Overall proportion incorrect (one to three dots of distractor groups selected) across participants for Experiment 2. Error bars denote standard errors of the means

The proportion correct data indicates that increased spacing within a group led to more difficulty tracking the group. But there remains an open question as to what is happening to the groups that participants are unable to track. Are whole groups being dropped from memory or is there confusion between parts of groups? We calculated the partial proportion of incorrect responses across each extent of inter-object spacing, defined as instances in which distractor groups in which some (one to three), but not all (four), dots were selected. An ANOVA revealed a main effect of inter-object spacing, F(3,111) = 54.90, p < 0.001, partial η^2^ = 0.60 (see Fig. 8), and proportion incorrect for partial groups was highest for the separate spacing (47.2%), which was significantly higher than the far spacing (M = 29.0%), t(37) = -4.82, p < 0.001. Proportion incorrect for partial groups was very low for near (>1%) and intermediate (M = 3.0%) spacings, and all conditions were significantly different from one another, *p*s < 0.05. These data suggest that at near and intermediate spacings, incorrect responses were due to confusion between entire groups, but for far and separate spacings, incorrect responding was driven by confusion between parts of groups.

Based on these findings, we know that both inter-object proximity and common fate are important cues for the perception of a group of objects. But to what extent does common fate have to be “complete”? Can individuated objects deviate from a group’s overall common fate, yet not disrupt the perception of a group? Experiment 3 investigated how the motion quality of a group impacts the extent to which objects are perceived as a group.

## Experiment 3

The third experiment investigated the role of common fate in perceptual grouping. Prior work has focused on motion trajectories of individual objects (Keane & Pylyshyn, 2006), motion trajectories of objects as they’re differentiated from distractors (Suganuma & Yokosawa, 2006), and motion trajectories of individuated objects that can be grouped (Merkel, Hopf, & Shoenfeld, 2017; Yantis, 1992). Here, we investigated the extent to which group objects can deviate from a group’s overall common motion, yet those objects still be perceived as a group. This design maintains group common fate, but individual objects within a group deviate from the group’s overall common fate. This allows for an investigation of the role of common fate at multiple levels. As such, in this experiment, there was a constant inter-object spacing and common fate for each group, but the groups were arranged in non-rigid formations. Individual objects randomly jittered at different eccentricities such that they spanned movement formations ranging from no jitter to relatively “high” jitter. This design allows us to investigate the role of common fate in the perception of dynamic groups of objects, particularly the extent to which group motion has to maintain motion unison. If group perception is contingent on complete common fate, then we would observe that tracking performance declines as jitter eccentricity increases.

### Participants

Forty participants (25 females; mean age = 22.98 years) participated in Experiment 3a and 41 participants (25 females; mean age = 21.05 years) participated in Experiment 3b. Six participants from Experiment 3b were excluded for having accuracy below 50% (mean dot accuracy = 34.9%).

### Stimuli and procedure

Experiments 3a and 3b consisted of movement eccentricity manipulations to examine the impact that common fate has on group perception and tracking. Stimuli and procedures for Experiments 3a and 3b were identical, with the exception that Experiment 3b consisted of expanded movement eccentricities (described in more detail below). Participants completed four blocks of 16 trials, yielding 64 total experimental trials. Four practice trials, representing movement eccentricities from each block, preceded the experimental trials.

**Fig. 9:**
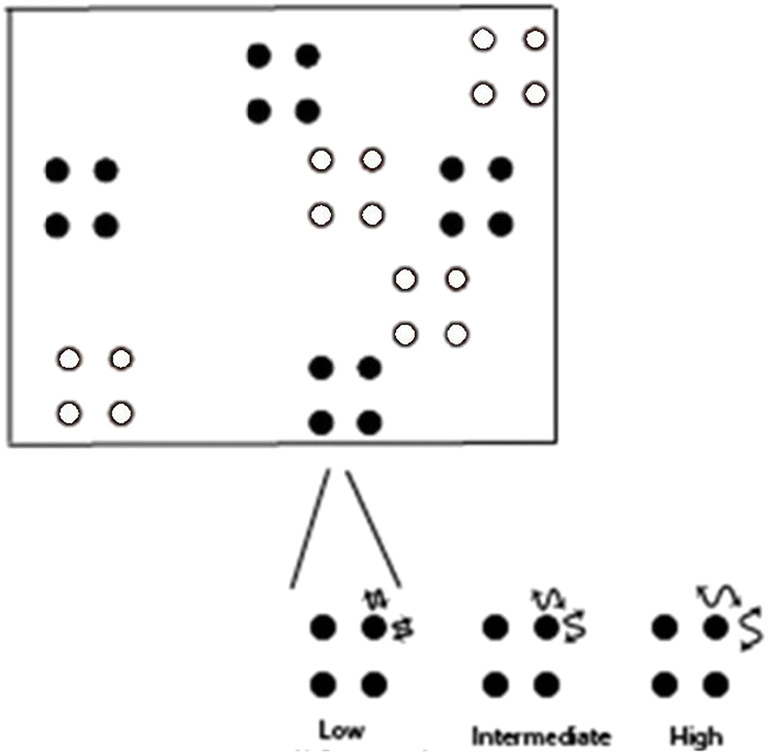
Stimuli for Experiments 3a and 3b. Experiment 3b consisted of a 40% increase in movement eccentricity. White-colored dots did not appear in the actual experiment but are used for representation of groups in the figure

The primary factor that varied across blocks was the movement eccentricity of the dots (see Fig. 9). While the groups of dots had a constant inter-object spacing across all blocks (which reflected the “intermediate” spacing in Experiment 2), the groups had non-rigid formations, such that the individual dots in each group formation randomly jittered within a prespecified eccentricity. Therefore, generation of group’s motion was similar to Experiments 1 and 2, with some variations. First, there was no constraint to the groups overlapping with one another. Second, across blocks of trials, the dots jittered at increasing eccentricities. There were four blocks: “no,” “low,” “intermediate,” and “high” jitter. In the no-jitter block, the groups of dots were rigidly formed, as in Experiment 2. In the other three blocks, the dots jittered within a given radius that was defined within a quadrant of an imaginary square enclosing the group formation. For Experiment 3a, the radii (in visual angle) for the jitter of the dots in each block were as follows: Low = 0.64^o^, Intermediate = 1.12^o^, High = 1.6^o^. For Experiment 3b, the radii (in visual angle) for the jitter of the dots in each block were as follows: Low = 1.12^o^, Intermediate = 1.76^o^, High = 2.4^o^.

Within each block, jitter eccentricity was identical for target and distractor groups. Like Experiment 2, the group size and number of target/distractor groups did not vary across blocks, such that all blocks consisted of groups composed of four dots, and participants were always required to track four target groups amongst four distractor groups. Dot size remained constant at 0.30^o^ radius across all blocks. Participants were asked to perform the tracking task and were explicitly told that the movement of the dots would change across blocks. Like Experiment 2, they were asked to select 16 dots at probe (four dots in each group x four groups). Targets were identified and tracking ensued in a manner identical to Experiments 1 and 2.

### Experiment 3: Results and discussion

**Fig. 10:**
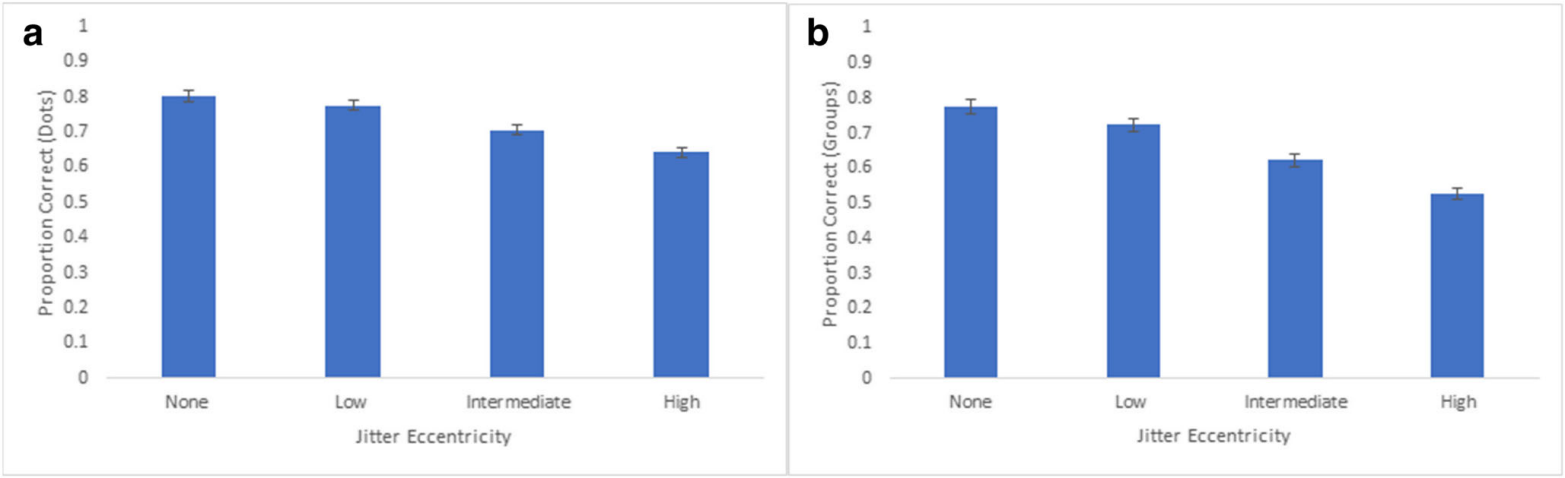
**a** Overall proportion correct (dots selected) across participants for Experiment 3a. Error bars denote standard errors of the means. **b** Overall proportion of correct groups (all four dots selected) across participants for Experiment 3a. Error bars denote standard errors of the means

**Fig. 11:**
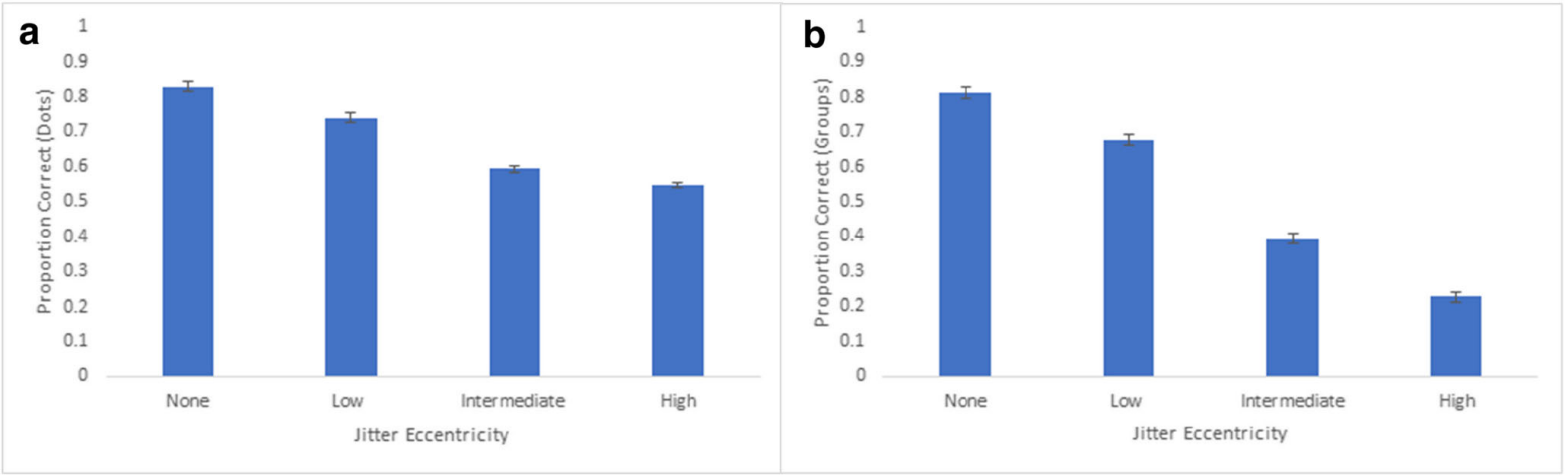
**a** Overall proportion correct (dots selected) across participants for Experiment 3b. Error bars denote standard errors of the means. **b** Overall proportion correct groups (all four dots selected) across participants for Experiment 3b. Error bars denote standard errors of the means. Note: Low-jitter eccentricity in Experiment 3b corresponds to Intermediate-jitter eccentricity in Experiment 3a

We calculated the average proportion of correct responses for each extent of jitter eccentricity (denoted as movement in the figures below) based on both dot and group responses. Dot accuracy was defined as the number of correctly selected dots on each trial, from a total of 16 dots. Group accuracy was defined as the number groups correctly selected in their entirety (i.e., four dots per group). For Experiment 3a, an analysis of variance revealed a main effect of movement for both dot accuracy, F(3,117) = 61.91, p < 0.001, partial η^2^ = 0.61, and group accuracy, F(3,117) = 100.27, p < 0.001, partial η^2^ = 0.72 (see Fig. 10). Similar patterns emerged for Experiment 3b, with a significant main effect of movement for both dot accuracy and F(3,102) = 230.92, p < 0.001, partial η^2^ = 0.87, and group accuracy, F(3,102) = 507.42, p < 0.001, partial η^2^ = 0.94^1^ (see Fig. 11). Generally, tracking performance gradually declined as jitter eccentricity increased, both in regards to comparisons across movement within each experiment, as well as comparisons between Experiments 3a and 3b. For Experiment 3b, group accuracy was highest for the no-jitter condition (M = 81.3%), which was significantly higher than the low-jitter condition (M = 67.7%), t(34) = 8.37, p < 0.001. Low-jitter group accuracy was significantly higher than intermediate-jitter group accuracy (M = 39.4%), t(34) = 14.71, p < 0.001, which was also significantly higher than the high-jitter group accuracy (M = 22.8%), t(34) = 13.76, p < 0.001.

**Fig. 12:**
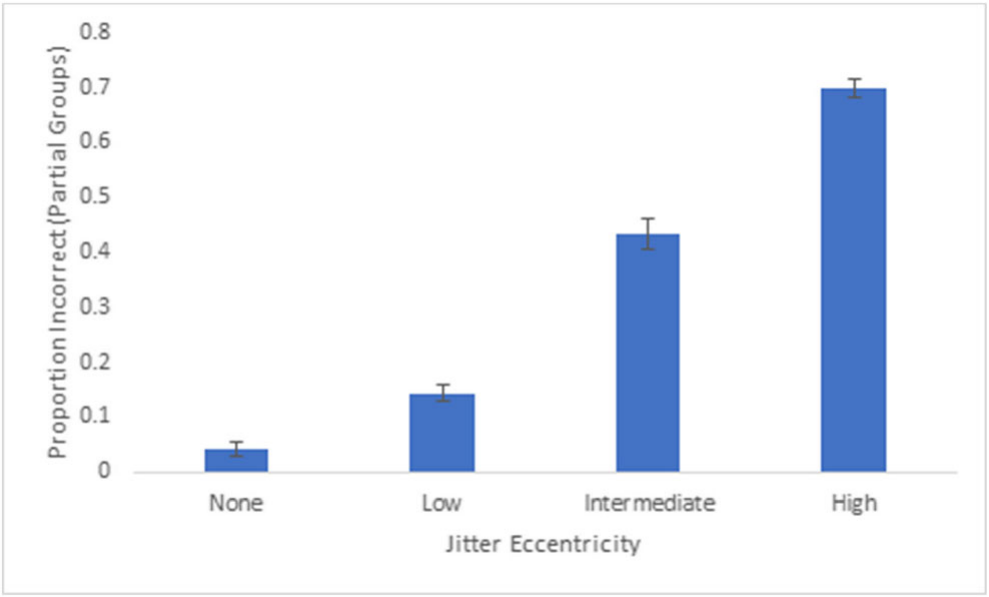
Overall proportion incorrect (one to three dots of distractor groups selected) across participants for Experiment 3b. Error bars denote standard errors of the means

We calculated the partial proportion of incorrect responses across each extent of jitter eccentricity to determine the extent to which there was confusion between parts of groups. This was defined as instances in which distractor groups in which some (one to three), but not all (four), dots were selected. An ANOVA revealed a main effect of movement, F(3,102) = 357.74, p < 0.001, partial η^2^ = 0.91 (see Fig. 12), and proportion incorrect for partial groups was highest for the high-jitter eccentricity (69.9.2%), which was significantly higher than the intermediate-jitter eccentricity (M = 43.4%). Proportion incorrect for partial groups was low for low- (M = 14%) and no- (M = 4.0%) jitter eccentricities, and all conditions were significantly different from one another, *p*s < 0.001. These data suggest that at no-movement and low-jitter eccentricities, incorrect responses were due to confusion between entire groups, but for intermediate- and high-jitter eccentricities, incorrect responding was more likely due to confusion between parts of groups.

Overall, we observe that more expanded movement eccentricities make groups of objects more difficult to track. This is based on the data that reveal a gradual decline in performance across increasing movement eccentricities. In addition, this held true in Experiment 3a, where movement eccentricities were more similar to one another, i.e., not largely discriminable, and also when movement eccentricities were more dramatic, as in Experiment 3b. Therefore, common fate is a powerful grouping cue, such that individual group objects can deviate from the overall common motion, yet group perception is maintained. However, there is a limit to this deviation, such that, when movement eccentricities are large and common fate is increasingly perturbed, the perception of a group of objects begins to deteriorate.

## General discussion

These experiments contribute to our understanding of grouping properties that impact multiple-object tracking. We investigated the role of group size, inter-object spacing, and common fate deviations to characterize the qualities that define ensembles when perceived in dynamic contexts. Across experiments we found tracking capacity estimates of approximately four groups of objects, regardless of the number of items a group was composed of. This is consistent with the pervasive finding of capacity limits of approximately three to five individuated items (Pylyshyn & Storm,^1988^) and suggests that groups of objects are perceived similarly to individual objects in the context of multiple-object tracking. However, it is also evident that the properties of groups are bound by heuristics of organization such that deviations to these organizational properties disrupt one’s ability to perceive and track a group of moving objects. Specifically, tracking performance declines as inter-object spacing increases and as individual objects deviate from a group’s common fate motion trajectory.

Based on these findings, group-based MOT operates similar to object-based MOT, given adherence to Gestalt principles of organization. This suggests the visual system’s use of an adaptive strategy for representation. This is in line with work conducted on the representations of multiple static objects, which suggests that sets of objects are represented as ensembles by relying on extracting average statistics of a group of objects (Alvarez, 2011). This strategy enables the visual system to cope with limitations to visual processing. Ensemble representation facilitates the system to use higher order representations for multiple objects, rather than relying on capacity to represent individuated items. This is implemented primarily through mechanisms of averaging across objects by building on the redundancy found in real-world images (Whitney & Yamanashi Leib, 2018). In sum, computations in the brain are made more efficient and are capable of supporting more expansive visual input, both that is static and dynamic.

As such, groups, within the context of MOT, are perceived as multi-part objects and are defined by proximity and common fate parameters. These parameters impact one’s ability to effectively track groups of objects, such that deviations in common fate motion and increased object spacing make tracking groups of objects more difficult. Groups of objects that have low inter-object spacing and follow common fate are more likely to be perceived as rigidly connected multi-part objects. These findings elucidate the need for more work on the perception of dynamic ensembles, as differences emerge between static and moving ensembles. Some work on static ensembles, specifically the perception of face ensembles, indicates that summary representations of facial expressions are immune to outliers (Haberman & Whitney, 2010). Of course, the analysis of emotional outliers belongs to a high-level domain and the computation of summary representations of facial expressions relies on the vast majority of stimuli present. Within other feature domains, such as brightness or orientation, the impact of outliers varies. Thus, delineating the differences in ensemble coding for static versus moving groups, as well as for different feature domains (both low and high level) is crucial. This work further contributes to our understanding of the mechanisms involved in the perception of groups of objects found in a wide variety of real-world settings.

Furthermore, the current investigation lends understanding to Gestalt theories as they apply to moving stimuli. Specifically, proximity (which we characterize as inter-object spacing) and common fate still stand as powerful grouping cues. Common fate is theoretically defined such that “all else being equal, elements that move in the same way, tend to be grouped together” (Wertheimer, 1923). Our data underscores the qualities of “equalness” and “moving the same way,” in that grouping weakens as sameness degrades. Also, the principle of common fate has been recognized to be applicable in a wide range of conditions, but how wide, has not been explicitly defined (Wagemans et al., 2012). Others have presented extensions to common fate grouping by examining common luminance changes (Sekuler & Bennett, 2001). They find that common fate not only operates for common motion of elements in physical space, but through luminance space as well. Here, we characterized common fate that operates on both a macro and a micro level. In our design, groups had an overall common fate trajectory, but individual objects did not, suggesting a hierarchical quality to grouping. As long as overall common fate is intact, grouping still occurs, but perturbations to overall common fate weakens its strength as a grouping cue. This is in line with Wagemans et al.’ (2012) suggestion of a more generalized common fate-grouping principle.

Numerous questions pertaining to the perception of multiple groups of objects remain unanswered. One area of investigation that could elucidate the nuances of group MOT is examining the role of multiple features in grouping perception. Specifically, what happens when more than one property of organization guides the perception of multiple groups of objects? For example, if comparing inter-object spacing and jitter eccentricity, does one property play a larger role in the perception of objects as a group? Also, examining the role of other salient features, such as color, and how they interact with the properties examined in the current investigation will help further characterize the boundaries of organization properties for groups of objects. Work by Emmanouil and Tresiman (2008) found that observers can perceive both the size and speed of a group of objects, but accuracy is lower for when there were multiple ensembles versus a single ensemble. Another area of inquiry should examine the perception of dynamic objects which exceed known capacity limits (i.e., four) to determine if the threshold for when tracking ability declines fluctuates as a function of the number of groups one is tracking. Work on static ensembles reports that observers can successfully extract multiple ensembles from up to four groups of stimuli (Attarha & Moore, 2015). Extending these investigations to moving ensembles will characterize humans’ ability to perceive and track multiple groups of objects in the real world.
